# Vitamin D Deficiency and Janus kinase 2 V617F Mutation Status in Essential Thrombocythemia and Polycythemia Vera

**DOI:** 10.21315/mjms2020.27.1.7

**Published:** 2020-02-27

**Authors:** Aysun Şentürk Yikilmaz, Sema Akinci, Şule Mine Bakanay, İmdat Dilek

**Affiliations:** Department of Hematology, Yıldırım Beyazıt University, Turkey

**Keywords:** vitamin D deficiency, chronic myeloproliferative neoplasia, polycythemia vera, essential thrombocythemia

## Abstract

**Introduction:**

Vitamin D, which is known for its effects on calcium and bone metabolism, has recently been associated with haematological malignancies. We aimed to investigate the relationship between disease findings and vitamin D deficiency in essential thrombocythemia (ET) and polycythemia vera (PV).

**Material and Methods:**

This retrospective cohort study conducted in Turkey included 73 patients diagnosed with PV or ET according to WHO criteria between 2012 and 2018. Vitamin D deficiency was defined as 25-OH vitamin D < 20 ng/mL. Polymerase chain reaction (PCR) was used to detect the Janus kinase 2 (JAK2) V617F mutation.

**Results:**

Vitamin D deficiency was found in 66.7% of PV and 74.2% of ET patients. The median follow-up time of ET and PV patients was 48 months and 47 months, respectively. Patients with the JAK2 mutation had a higher prevalence of a history of thrombosis and age older than 65 years. There was a significant relationship between JAK2 positivity and vitamin D deficiency.

**Conclusion:**

There was a remarkably higher prevalence of vitamin D deficiency in JAK2 mutation-positive ET and PV patients. These patients should be carefully evaluated for vitamin D deficiency. More studies are required to further investigate the association between JAK2 and vitamin D.

## Introduction

Chronic myeloproliferative neoplasms (CMPNs) are diseases of clonal multipotent stem cells characterised by uncontrolled proliferation involving the three hematopoietic series in the bone marrow. There are four classical subgroups: chronic myeloid leukemia (CML), primary myelofibrosis (PMF), polycythemia vera (PV), and essential thrombocythemia (ET). Hypersensitivity to cytokines and growth factors, such as insulin-like growth factor 1 (IGF-1), interleukin-13, granulocyte-macrophage colony-stimulating factor (GM-CSF), stem cell factor, thrombopoietin and resistance to apoptosis in progenitors play a role in the pathogenesis of CMPNs (1) as well as somatic mutations, such as the Janus kinases-signal transducers and activators of transcription (JAK-STAT) signaling pathway, Janus kinase 2 (JAK2) tyrosine kinase, calreticulin (CALR), which is the gene for the calcium binding protein calreticulin and myeloproliferative (MPL), which encodes the thrombopoietin receptor (2, 3). The single acquired JAK2 V617F point mutation has been described in almost all patients with PV and 60%–65% of patients with ET (2). JAK2 is an effective gene for tyrosine kinase found on the short arm of chromosome 9. In CMPN cases, JAK2 mutations have been shown to cause hypersensitivity in hematopoietic progenitor cells towards growth factors and other cytokines (2). The presence of JAK2 mutations in erythropoietin (EPO)-independent erythroid colonies correlates this mutation with growth factor hypersensitivity. Further investigation of the JAK2 and STAT pathway may provide a more reliable classification/diagnostic system for CMPNs and a better understanding of their pathophysiology. Patients with CMPNs may experience major complications such as thrombosis, bleeding and transformation to acute myeloid leukemia or myelofibrosis. Despite serious complications, the management of CMPN patients with modern treatment methods has increased the life expectancy (4, 5).

The effects of 25-hydroxyvitamin D [25(OH) D] on calcium and phosphate metabolism and bone balance are well known. However, it can also play a role in cell proliferation, differentiation, and cell adhesion as well as proliferation and apoptosis of tumour cells. Additionally, it modifies tumour angiogenesis, invasion and metastasis with reduced oxidative DNA damage (6). Studies have been published on the association between vitamin D and the JAK-STAT pathway. (**7**).

The main source of vitamin D is transformation from 7-dehydrocholesterol to vitamin D3 (cholecalciferol) by endogenous ultraviolet B-rays in the skin via the photochemical pathway (8). Dietary vitamin D can be ingested as ergocalciferol (vitamin D2) from plants or cholecalciferol (vitamin D3) from animal tissues. Dietary or endogenous vitamin D2 or vitamin D3 is stored in fat cells and released into the circulation when necessary (8). Vitamin D produced in the skin or ingested from the diet is biologically inactive (8). It is first converted to 25(OH)D with 25-hydroxylase enzymes in the liver and then, with the 1-alpha hydroxylase enzyme to 1,25-dihydroxyvitamin D [1,25(OH)2D], also known as calcitriol, which is biologically active in the kidneys (8). To assess vitamin D levels, the level of 25(OH)D, which has a half-life of 2–3 weeks and indicates both vitamin D intake and endogenous production, should be evaluated (8). The biologically active form [i.e., 1,25(OH)2D] is not ideally suited for measurement (8) because its half-life is as short as 4–6 h and its circulating levels are 1,000 times lower than those of 25(OH) D (9). Many studies have been conducted to identify vitamin D deficiency and to determine the normal range of 25(OH)D levels. In scope of these studies, vitamin D deficiency was accepted as 25(OH)D levels of < 20 ng/mL (9). Measurement of serum 25(OH)D levels is an indirect way to determine vitamin D levels in patients (8). The Endocrine Society also defines vitamin D deficiency as 25(OH)D serum levels < 20 ng/mL, but it is unclear exactly which vitamin D levels are required to maintain a healthy lifespan (9). Although vitamin D deficiency is common worldwide in the general healthy population, the evolution and prognosis of some cancers have been associated with vitamin D deficiency (10). A meta-analysis of seven studies showed that low serum 25(OH)D levels were strongly associated with shortened overall survival and recurrence-free survival in 2,643 patients with haematological cancer (11).

In a study on patients with chronic myeloproliferative neoplasia, ET patients and PV patients were compared according to vitamin D levels and they found that vitamin D deficiency was more prevalent in PV patients (12). Therefore, there is a remarkable association between 25(OH)D levels and state of disease state in various haematological cancers.

This study aimed to determine whether there is a difference in vitamin D deficiency between ET and PV patients and to investigate the relationship between vitamin D deficiency and clinical-laboratory findings in these groups.

## Material and Methods

### Patients

The study was conducted at the Hematology Department of Ankara Atatürk Training and Research Hospital between 2012 and 2018. Patients diagnosed with ET and PV older than 18 years of age were included in the study. PV and ET diagnoses were based on the 2016 WHO diagnostic criteria (13). A bone marrow biopsy was performed in all patients who were diagnosed according to 2013 WHO criteria and the diagnosis was confirmed according to 2016 WHO criteria. PMF cases were not included due to the low number of cases. Clinical and laboratory findings, 25(OH)D levels at the initial diagnosis and complications experienced by patients were obtained from patient records and retrospectively evaluated.

According to the 2016 WHO criteria, PV diagnosis requires all three major criteria or the first two major criteria and one minor criterion must be positive (13). All criteria are defined below.

Major criteria:

- Haemoglobin > 16.5 g/dL or hematocrit > 49% in men, haemoglobin > 16.0 g/dL or hematocrit > 48% in women or increased red cell mass (> 25% above mean normal predicted value)
- Bone marrow (BM) biopsy showing hypercellularity for age with trilineage growth (panmyelosis) including prominent erythroid, granulocytic and megakaryocytic proliferation with pleomorphic, mature megakaryocytes (differences in size)
- Presence of JAK2 or JAK2 exon 12 mutations

Minor criteria:

- Subnormal serum EPO level
  ET diagnosis requires all four major criteria or the first three major criteria and one minor criteria (according to 2016 WHO criteria) (13). All criteria are defined below.

Major criteria:

- Platelet count ≥ 450 × 10^9^/L
- BM biopsy showing proliferation mainly of the megakaryocyte lineage with increased numbers of enlarged, mature megakaryocytes with hyperlobulated nuclei, no significant left-shift of neutrophil granulopoiesis or erythropoiesis and very rarely, a minor (grade 1) increase in reticulin fibers (grading of BM fibers).
- Not meeting WHO criteria for BCR-ABL (t[9;22] chromosomal translocation)1+ CML, PV, PMF, myelodysplastic syndrome (MDS) or other myeloid neoplasms.
- Presence of JAK2, CALR or MPL mutations.

Minor criteria:

- Presence of a clonal marker (e.g., abnormal karyotype) or absence of evidence for reactive thrombocytosis.

### Laboratory Tests

Plasma levels of 25(OH)D, which are frequently used to determine vitamin D status, were measured in 10 mL peripheral blood samples at initial diagnosis. The 25(OH)D levels were measured with high performance liquid chromatography. Levels < 20 ng/mL were considered to indicate vitamin D deficiency.

The JAK2 V617F mutation was determined with real time PCR using 10 mL peripheral blood samples at initial diagnosis. Real time PCR is a technique used to determine and quantify DNA in real time using fluorescent dyes. The fluorescence signal increases in proportion to the amount of PCR product. The results can be obtained during PCR analysis.

### Statistical Analysis

Normally distributed variables are expressed as mean (SD) and non-normally distributed variables are expressed as median (IQR). Categorical variables such as gender, diagnosis status and thrombosis history are indicated by frequency (%). The Mann-Whitney U test was used to compare continuous variables according to the presence of the JAK2 V617F mutation and treatment status. Gender and vitamin D levels in the groups were evaluated with Chi-squared tests. The level of statistical significance was set at *P* < 0.05. For statistical analysis and calculations, IBM SPSS Statistics 21.0 (IBM Corp. released 2012. IBM SPSS Statistics for Windows, version 21.0. Armonk, NY: IBM Corp.) was used.

## Results

A total of 73 (42 PV, 31 ET) CMPN patients were studied. The female/male ratio was 18 (42.9%)/24 (57.1%) in PV patients and 16 (51.6%)/15 (48.4%) in ET patients. The median age of the patients was 60.3 (IQR 30–83) years in PV patients and 58.8 (IQR 29–80) years in ET patients. Table 1 summarises the laboratory and clinical parameters of the ET and PV patient population at the time of diagnosis. Vitamin D deficiency was found in 28 (66.7%) of the PV patients and 23 (74.2%) of the ET patients. A history of thrombosis was present in 6 (14.2%) PV patients and 4 (12.9%) ET patients. All patients received acetylsalicylic acid. In total, 28 (66.7%) PV patients and 23 (74.2%) ET patients were treated with hydroxyurea; 12 (%16.4) PV patients were managed with phlebotomies. The median follow-up period of ET patients and PV patients was 48 (IQR 20–96) months and 47 (IQR 13–158) months, respectively. The JAK2 V617F mutation had a significantly higher prevalence in patients over 65 years of age (*P* = 0.002). Thrombosis was more frequent in patients with the JAK2 V617F mutation (*P* = 0.034). There was no significant difference in 25(OH)D deficiency between the ET or PV groups (*P* = 0.608).

The relationship between clinical/laboratory findings and 25(OH)D levels was evaluated for each group. The relationship between the findings in PV patients and 25(OH)D levels is shown in Table 2. There was a significant relationship between JAK2 V617F mutation positivity and vitamin D deficiency in PV patients (*P* = 0.024). There was also a significant correlation between hydroxyurea use and vitamin D deficiency (*P* = 0.005).

Table 2 summarises the comparisons between the clinical/laboratory findings and 25(OH)D levels in ET patients. There was a significant association between low levels of 25(OH)D and the JAK2 V617F mutation in ET patients (*P* = 0.011).

## Discussion

Vitamin D deficiency is present in approximately 30%–50% of the world’s general population and is a common health problem (9). In this study, vitamin D deficiency was present in 66.7% of PV patients and 74.2% of ET patients. This rate was slightly higher than expected compared to the general healthy population. However, there is a limited number of studies on vitamin D levels in CMPD. One study on CMN patients, including 63 PV and 25 ET patients, demonstrated that vitamin D deficiency was slightly more frequent in PV patients (62%) than in ET patients (43%) (12). Our study demonstrated a similar frequency in both groups at 66.7% in PV and 74.2% in ET patients. Although a healthy control population would have been more appropriate for comparison, the effect of disease itself on vitamin D deficiency cannot be ruled out. Studies in both healthy and patient populations in different parts of the world, including Turkey, have shown that vitamin D deficiency is more common in women (14–17). However, among patients with ET or PV, the prevalence of vitamin D deficiency was similar between female and males in our study. In addition to female gender, elderly age and living in a nursing home may also increase the risk of vitamin D deficiency.

In our study, vitamin D deficiency was more frequent in JAK2 V617F-positive PV and ET patients. Almost all PV cases usually have JAK2 V617F mutations, while 60%–65% of ET patients have mutation. Although vitamin D deficiency was more common in both groups with the JAK2 V617F mutation, more comprehensive studies on the relationship between JAK2 V617F mutation positivity and vitamin D should be conducted. There are studies in the literature that suggest a relationship between the JAK-STAT pathway and vitamin D deficiency (7). Many studies report that 95%–97% of PV patients have a JAK2 V617F mutation (18). However, the presence of the JAK2 V617F mutation is not specific for PV because it is also found in a significant proportion of patients with ET and PMF (18). Mutant JAK2 can bind to any receptor or phosphorylation STATs in patients that have little or no EPO (18). Therefore, uncontrolled hematopoiesis may be induced. In our study, PV patients had a higher prevalence of the JAK2 V617F mutation compared to ET patients, as expected, and the JAK2 V617F mutation rate in ET patients was consistent with that in the literature. In a previously published experimental study on mice, the binding of vitamin D and its receptor was shown to suppress JAK/STAT pathway-mediated T cell differentiation and cytokine production in the spleen (19). Vitamin D can inhibit inflammation by suppressing cytokine signals and its production. The JAK-STAT signal is required for the proliferative effects of many cytokines, including the pro-inflammatory cytokines IL6, IL12 and IFN gamma (20). In a study of patients with prostate cancer, 1,25(OH)D was found to suppress many JAK-STAT signal components, including JAK1, STAT1, STAT2 and STAT3, which is consistent with our study (21). It has been suggested that the prominent anti-inflammatory effect of vitamin D in prostate epithelial cells occurs via suppression of the JAK-STAT signaling pathway. Many studies have been conducted to determine any association between various disease states and vitamin D deficiency. In a study consisting of cases of experimental allergic encephalomyelitis, an autoimmune disease of the central nervous system, an inhibitory effect of 1,25(OH)2D on JAK signal pathways was observed (22). In another publication, vitamin D stimulated a prolonged effect of growth hormone by suppressing the JAK2 signaling pathway in osteoblast-like cells (23).

Vitamin D deficiency was also associated with a decrease in total survival and/or PFS for acute myeloid leukemia, CLL and NHL (24–26). In a study with 34,763 women with a median age of 63 years, moderate calcium replacement was shown to prevent lymphoid malignancies in elderly women (27). In our patient group, we could not find a relationship between complications, such as thrombosis, haemorrhage, erythrocytosis, thrombocytosis, leukocytosis, and vitamin D deficiency, but the number of patients was insufficient to make a solid conclusion.

Further comprehensive studies are required to elucidate the relationship between vitamin D deficiency and the JAK2 V617F mutation in this patient population. It is important to clarify whether vitamin D replacement therapy can affect JAK positivity, one of the major clonal mutations in this patient group.

## Conclusion

Vitamin D deficiency is a common health problem among various populations worldwide. According to our study, there was a high prevalence of vitamin D deficiency in patients with PV and ET. JAK2 V617F mutation-positive PV and ET patients had lower vitamin D levels.

## Figures and Tables

**Table 1:** Clinical and laboratory findings in PV and ET patients

	PV patients *n* = 42 (57.5%)	ET patients *n* = 31 (42.5%)	*P*-value
Age (years), median (IQR)	60.3 (30–83)	58.8 (29–80)	0.782
Age > 65 years, *n* (%)	17 (40.5%)	11 (36.7%)	0.809
Gender (female/male)	18 (42.9%)/24 (57.1%)	16 (51.6%)/15 (48.4%)	0.634
Haemoglobin (g/dL), median (IQR)	17.6 (11.9–21.3)	14.1 (11.5–19.4)	**0.001**
Leukocytes (count × 10^6^/L), median (IQR)	12577 (1760–28900)	11718 (2930–30500)	0.140
Lymphocytes (count × 10^6^/L), median (IQR)	2326 (750–10150)	2385 (640–5290)	> 0.95
Basophils (count × 10^6^/L), median (IQR)	105.6 (3–500)	118 (5–900)	0.061
Thrombocytes (count × 10^6^/L), median (IQR)	430000 (155000–1601000)	773000 (255000–2130000)	**0.001**
LDH (g/dL), mean [median (IQR)]	325 (196–597)	311 (206–736)	0.802
Sedimentation (mm/h), median (IQR)	8 (1–49)	19 (1–98)	0.054
Thrombosis			
Present	6 (14.2%)	4 (12.9%)	0.302
Absent	36 (85.8%)	27 (87.1%)	
Haemorrhage			
Present	2 (4.8%)	4 (12.9%)	0.227
Absent	40 (95.2%)	27 (87.1%)	
JAK2 V617F status			
Positive	31 (73.8%)	17 (54.8%)	**0.026**
Negative	11 (26.2%)	15 (45.2%)	
Treatment			
Hydroxyurea positive	28 (66.7%)	23 (74.2%)	0.436
Hydroxyurea negative	14 (33.3%)	8 (25.8%)	
Acetylsalicylic acid	42 (100%)	31 (100%)	> 0.95
Phlebotomy	12 (28.5%)	0 (0%)	**0.001**
Vitamin D			
< 20	28 (66.7%)	23 (74.2%)	0.608
≥ 20	14 (33.3%)	8 (25.8%)	
Total 25(OH)D (ng/mL), median	15.2 (6–60)	13.3 (2–97)	0.701

**Table 2:** Comparison of clinical and laboratory findings according to vitamin D levels in PV and ET patients

	PV patients			ET patients		

	Deficient vitamin D *n* = 28 (66.7%)	Normal vitamin D *n* = 14 (33.3%)	*P*-value	Deficient vitamin D *n* = 23 (74.2%)	Normal vitamin D *n* = 8 (25.8 %)	*P*-value
Age (≤ 65/ > 65)	15 (35.7%)/13 (31%)	10 (23.8%)/4 (9.5%)	0.330	16 (51.4%)/4 (12.9%)	7 (22.6%)/4 (12.9%)	0.405
Gender (male versus female)	18 (42.9%)/10	6 (14.3%)/(19%)	0.208	13 (41.9%)/10 (32.3%)	2 (6.5%)/6 (19.4%)	0.220
Haemorrhage	1 (2.4%)/27 (64.3%)	1 (2.4%)/13 (31%)	1.000	3 (9.7 %)/20 (64.5 %)	1 (3.2%)/7 (22.6 %)	> 0.95
Thrombosis (+/−)	4 (9.5%)/24 (57.%)	0 (0%)/14 (33.3%)	0.283	6 (19.4%)/17 (54.8%)	0 (0%)/8 (25.8%)	0.298
JAK 2 V617F Status (+/−)	24 (57.1%)/4 (9.5%)	7 (16.7%)/7 (16.7%)	**0.024**	16 (51.6%)/7 (22.6%)	7 (22.6%)/1 (3.9%)	**0.008**
Treatment (Hydroxyurea +/ Hydroxyurea −)	23 (54.8%)/5 (11.9%)	5 (11.9%)/9 (21.4%)	**0.005**	15 (48.4%)/8 (25.8%)	8 (25.8%)/0 (0%)	0.057
